# Response to: ‘Lack of evidence for Zika virus transmission by *Culex* mosquitoes’

**DOI:** 10.1038/emi.2017.86

**Published:** 2017-10-18

**Authors:** Constância Ayres, Duschinka Guedes, Marcelo Paiva, Mariana Donato, Priscilla Barbosa, Larissa Krokovsky, Sura Rocha, Karina Saraiva, Mônica Crespo, Tatiana Rezende, Gabriel Wallau, Rosângela Barbosa, Cláudia Oliveira, Maria Alice Melo-Santos, Lindomar Pena, Marli Cordeiro, Rafael Franca, André Oliveira, Christina Peixoto, Walter S Leal

**Affiliations:** 1 Instituto Aggeu Magalhães (IAM), Fundação Oswaldo Cruz (Fiocruz). Av. Moraes Rego, s/n Campus da UFPE, Cidade Universitária, Recife, PE 50670-420, Brazil; 2 Centro Acadêmico do Agreste, Universidade Federal de Pernambuco, Caruaru, PE 55002-970, Brazil; 3 University of California-Davis, Davis, CA 95616, USA

In response to the letter “Lack of evidence for Zika virus transmission by *Culex* mosquitoes” that questioned the findings published by Guedes *et al.*,^1^ we vehemently disagree with the overall perspective. The argument raised by the authors is flawed. First and foremost, none of the previous reports (published before the 2015 ZIKV epidemic) indicating *Aedes* species as natural ZIKV vectors;^2, 3, 4, 5, 6^ and the one that led to the incrimination of *Ae. aegypti* as the main vector,^7^ triaged the specimens by gonotrophic stages (blood fed, black blooded, gravid or unengorged). In all cases, the mosquitoes were collected, and virus isolation/detection was conducted regardless of the feeding status. Please note that our results provide more detailed data (mosquito feeding status) than the previous studies, because we considered it important regarding another potential vector. Therefore, our findings do also satisfy the third vector incrimination criterion.

Second, the only technical criticism of our study regarding the high Ct values that were reported for the field-collected samples is equally unfounded. The authors stated, ‘For ZIKV transmission in the Americas, criterion 3 has been met only for *Ae. aegypti*, with detection of naturally infected mosquitoes with titers compatible with transmission-competence in Mexico^8^ and Brazil.’^9^ However, the study in Mexico did not report any Ct value for the field-caught mosquito samples, the authors of this study only discussed the low titer levels observed for the *Ae. aegypti* field samples, and Ferreira-de-Brito *et al.*^9^ reported Ct values for *Ae. aegypti* (36.68, 37.78 and 38.04) that are very similar to those observed in our study for *Cx. quinquefasciatus*, although these authors were not able to isolate ZIKV. This suggests that ZIKV infection in nature may exhibit a different performance when compared with artificial infection under laboratory conditions. All other quoted papers where low Ct values were registered relate to controlled experimental infections and not to natural infections. Indeed, vector competence is only one parameter of vectorial capacity; which include other entomological traits, such as abundancy, longevity and biting rate, which are affected by abiotic factors.^10^ Therefore, the process of arbovirus transmission in nature is a complex phenomenon that should not be limited to previous simple models without considering the great capacity for virus evolution and adaptation to new hosts as well as the high genetic diversity observed within and between mosquito populations. Differences in results of vector competence studies are common, and negative results have also been reported for *Ae. aegypti*, as pointed out by Guedes *et al.*^1^ Unfortunately, the only study that has examined the same virus and *Culex* mosquito population reported very limited methodological details,^11^ making it impossible to compare both studies.

It is important to highlight that the first studies of ZIKV vectors were performed in the context of yellow fever surveillance, which led to a strong bias for *Aedes* species. If other species are also involved in ZIKV transmission, research should be conducted in an appropriate manner to consider different densities of mosquitoes present in Zika outbreak areas, including those areas where *Ae. aegypti* is absent. Unfortunately, these studies have not considered the high abundance of *Cx. quinquefasciatus*, and the sampling methods used in most of these studies have favored *Aedes* species (Table 1). This fact would explain the lack of evidence supporting the incrimination of *Culex* in ZIKV transmission as noted by the letter. Considering the abundance of *Cx. quinquefasciatus* in Recife, the analysis of a greater number of *Cx. quinquefasciatus* pools enabled the detection of ZIKV in this mosquito species.^1^

Last, although we were able to photograph mature ZIKV particles in the salivary glands at 7 days post infection, detected ZIKV-infected mosquitoes in nature, isolated ZIKV strains and sequenced the ZIKV genome from this mosquito species for the first time, which corroborates earlier finding,^14^ we understand that additional studies are needed to unambiguously establish the role of *Cx. quinquefasciatus* in ZIKV transmission. Additional reports concerning new data of virus surveillance in field-caught *Culex* and *Aedes* mosquitoes from other regions of Brazil are under review.

Regarding caution from part of the media and public health authorities, we followed the WHO statement on data sharing of relevant information,^15^ as mentioned in reference.^9^ We should also exercise caution not to overlook other potential vectors. After all, when comes to human health, caution is a two-way street. We believe that human population has the right to know about other possible routes of ZIKV transmission and to attempt preventing mosquito bites and thus reducing the risk of ZIKV infection.

To conclude, we would like to state that any researcher who is interested in repeating our experiments is very welcome at the Department of Entomology at Aggeu Magalhães Institute, Oswaldo Cruz Foundation. We believe that a healthy debate about Zika vectors will only contribute to the progress of scientific knowledge.

## Figures and Tables

**Table 1:** Number of female mosquitoes analyzed for ZIKV detection in recent outbreaks of Zika virus in the Americas

**Local**	***Aedes aegypti***		***Culex quinquefasciatus***		**References**
	***N***	**+**	***N***	**+**	
Rio de Janeiro, Brazil	315	2	385	0	^9^
Rio de Janeiro, Brazil	406	2	0	0	^12^
Recife, Brazil	408	2	1496	3	^1^
Chiapas, Mexico	472	15	∼151	0	^8^
Jalisco, Mexico	179	6	115	15	^13^

Abbreviations: Number of females tested, *N*; number of ZIKV-positive pools, +.
